# Radiation in Early-Stage Breast Cancer: Moving beyond an All or Nothing Approach

**DOI:** 10.3390/curroncol30010015

**Published:** 2022-12-23

**Authors:** Juhi M. Purswani, Camille Hardy-Abeloos, Carmen A. Perez, Maryann J. Kwa, Manjeet Chadha, Naamit K. Gerber

**Affiliations:** 1 Department of Radiation Oncology, NYU Grossman School of Medicine, New York, NY 10016, USA; 2 Department of Medical Oncology, NYU Grossman School of Medicine, New York, NY 10016, USA; 3 Department of Radiation Oncology, Icahn School of Medicine at Mount Sinai, New York, NY 10029, USA

**Keywords:** early-stage breast cancer, radiotherapy omission, partial breast irradiation, APBI, radiotherapy de-escalation

## Abstract

Radiotherapy omission is increasingly considered for selected patients with early-stage breast cancer. However, with emerging data on the safety and efficacy of radiotherapy de-escalation with partial breast irradiation and accelerated treatment regimens for low-risk breast cancer, it is necessary to move beyond an all-or-nothing approach. Here, we review existing data for radiotherapy omission, including the use of age, tumor subtype, and multigene profiling assays for selecting low-risk patients for whom omission is a reasonable strategy. We review data for de-escalated radiotherapy, including partial breast irradiation and acceleration of treatment time, emphasizing these regimens’ decreasing biological and financial toxicities. Lastly, we review evidence of omission of endocrine therapy. We emphasize ongoing research to define patient selection, treatment delivery, and toxicity outcomes for de-escalated adjuvant therapies better and highlight future directions.

## 1. Introduction

The multimodal treatment of early-stage breast cancer includes breast-conserving surgery (BCS), radiation therapy (RT), and endocrine therapy (ET). As patients with breast cancer are living longer, it becomes increasingly important to minimize treatment-related morbidity. As such, there has been growing interest in the de-escalation of radiotherapy for very favorable-risk breast cancer. While there are ongoing trials evaluating the omission of radiotherapy, there are evolving data demonstrating decreased biological and financial toxicity of accelerated partial breast irradiation. The purpose of this article is to describe the strategies for de-escalated adjuvant therapies in early-stage breast cancer, including radiation omission, accelerated partial breast irradiation, and the role of endocrine therapy for very low-risk hormone receptor-positive (HR+) breast cancer. 

## 2. Selecting Patients for Radiation Omission

### 2.1. Age as a Selection Criterion 

The concept of omission of RT for low-risk breast cancer dates to the early 1980s. At that time, the seminal NSABP B-06 trial had established the importance of RT in reducing the risk of an ipsilateral in-breast tumor recurrence (IBTR) after lumpectomy in women with node-positive or node-negative breast cancer [1]. Results from many clinical trials and EBCTCG meta-analysis established the benefit of adjuvant radiation therapy in reducing both IBTR and breast cancer deaths [2], as well as the benefit of endocrine therapy [3,4,5] in the treatment of early-stage HR+ breast cancer. The widely accepted recommendation for HR+ breast cancer includes five years of tamoxifen (TAM) [5] or an aromatase inhibitor (AIs) [4]. However, with the increasing use of mammography resulting in the detection of occult tumors, and data demonstrating the benefit of TAM in women with HR+ breast cancers, many began to question whether the benefit of RT observed in NSABP B-06 would apply to clinically low-risk patients. Key randomized controlled trials subsequently designed to evaluate clinical outcomes after the omission of RT in early-stage HR+ breast cancer are included in Table 1. 

The NSABP B-21 study opened in 1989 and randomized 1009 women with tumors ≤1 cm to TAM alone, RT, or RT + TAM [3]. There was a substantial risk of an IBTR after lumpectomy in patients with small tumors ≤1 cm despite TAM (16.5% at 8 years of follow-up versus 2.8% in the RT + TAM group). TAM alone was less effective than RT alone in reducing IBTRs. A Canadian trial on 769 women with pT1-2, N0 breast cancer from 1992–2000 randomized women to TAM alone or TAM + RT and reported an 8-year IBTR of 17.6% versus 3.5%, respectively [6]. In a planned subgroup analysis of T1, HR+ women, the 8-year IBTR was 15.2% versus 3.6%. In an unplanned subgroup analysis restricted to women with tumors ≤1 cm, HR+, and age ≥60, there was no difference in the rate of local relapse at 5 years (1.2% vs. 0%) or 8 years (3.6% vs. 0%) [7], suggesting that age could be used as a criterion to select patients for omission.

In two subsequent modern randomized control trials, age was used as a criterion for omission after breast-conserving surgery (BCS) in patients with HR+ disease undergoing ET. In the CALGB 9343, 636 women ≥70 with clinical stage I, ER+ breast cancer, were randomized to TAM or RT + TAM [8]. At 10 years, the locoregional recurrence-free survival was 98% in the RT + TAM arm versus 90% in the TAM arm. This did not translate into differences in OS, DFS, or breast preservation. Among the patients who died, the causes of death were largely unrelated to breast cancer. In the PRIME II study, 1326 women ≥65 with node-negative, ER+ breast cancer were randomized to RT versus no RT [9]. All patients received ET. At 10 years, the IBTR rate was 0.9% versus 9.8% in the RT and no RT arms, respectively (*p* = 0.0008). However, there were no differences in regional recurrences, contralateral breast cancer, or distant metastases. These data suggest that the absolute risk of recurrence is lower when selecting patients of older age, with most dying of non-breast cancer-related causes. As a result, the National Comprehensive Cancer Networks Guidelines included adjuvant ET alone as a category one recommendation for women >70 years of age with early-stage HR+ breast cancer.

Of note, despite modest absolute reductions in IBTR in PRIME II and NSABP B-21, the hazard ratio for the benefit of RT was similar (0.12 in PRIME II versus 0.19 in NSABP B21) and validated a persistent benefit of RT even in these early-stage breast cancer populations. The omission of RT based solely on age and HR status alone may lack appropriate risk-stratification power. It remains critical to identify a patient population with “low enough” IBTR risk following lumpectomy, for whom adjuvant RT may not provide a convincingly meaningful benefit. 

### 2.2. Combining Age and Biology 

Does age remain an important criterion for RT omission once we account for tumor subtype? In a retrospective study of 1434 patients who underwent breast-conserving therapy from 1997–2006, the crude risk of locoregional recurrence (LRR) varied drastically by tumor subtype [10]. Patients with luminal A (ER+/PR+/HER2-) and luminal HER2+ breast cancer had a 1.5% and 1.0% risk for local recurrence at a median follow-up of 85 and 82 months, respectively. In patients with the luminal B subtype, the risk rose to 4% at a median follow-up of 96.3 months. In patients with HER2+ and triple-negative subtypes, the risk rose to 10.9% and 8.8% at a median follow-up of 83.8 and 61.2 months, respectively. In young women, the luminal B and HER2 subtypes were associated with high rates of LR after BCS and RT, though increasing age was associated with decreased risk of LR independent of the breast cancer subtype. 

In the Canadian study discussed above, investigators developed a low-risk clinical model of patients with T1, grade 1 or 2 breast cancer and ≥60 years, and reported a 10-year IBTR rate of 4.6% compared to 13.7% for the high-risk clinical group [11]. Combining the low-risk model with the luminal A subtype, defined by IHC and proliferative index criteria, resulted in a 10-year IBTR rate of 1.3% with TAM compared to 5% with RT + TAM. On multivariable analysis, treatment with RT + TAM versus TAM alone, the intrinsic subtype and clinical risk group were significantly associated with IBTR. The LUMINA trial is a prospective multicenter cohort study of patients ≥age 55 with grade 1–2, T1N0 breast cancer status, post-lumpectomy with ≥1 mm margins and luminal A subtype (ER ≥ 1%, PR >20%, HER2 negative and ki-67 ≤13.25%). All patients are treated with adjuvant endocrine therapy (ET), and RT is omitted following BCS. Preliminary results from 501 patients were reported at the ASCO 2022 annual meeting. The median age was 67 years, with 88% of patients <75 years of age, and the median tumor size was 1.1 cm. At 5 years, the local recurrence rate was low at just 2.3%. 

Moving beyond IHC, gene signatures based on multigene profiling assays are increasingly being used as predictive and prognostic markers for breast cancer recurrence and have been incorporated into ongoing clinical trials for the omission of RT (Table 2). The PAM50 assay is a standardized 50-gene set for intrinsic subtype classification used for risk stratification. This assay is currently being investigated for the biological selection of low-risk breast cancers in the PRECISION and EXPERT trials. The Oncotype DX is a 21-gene classifier containing the mRNA quantification of ER, PR, HER2, and tumor proliferation that provides a recurrence score (RS) to quantify the risk of distant recurrence. In a study investigating the association between RS and LRR in node-negative and ER+ breast cancer from two NSABP trials, NSABP B-14 (TAM treated: *n* = 895, placebo-treated: *n* = 355) and NSABP B-20 (*n* = 424), RS was found to be a significant predictor of LRR in TAM treated, placebo treated and chemotherapy + TAM treated patients. Of note, only 2% of patients enrolled in the NSABP 20 were over the age of 70 years, and no patients over the age of 70 years were enrolled in NSABP B14. On multivariable analysis, RS, age, and type of treatment were independent predictors of LRR. DEBRA is a phase III trial that randomizes patients between 50 to 70 years of age, with HR+ early-stage breast cancer, and with an Oncotype DX RS ≤18 to adjuvant RT versus observation. 

We await results from these prospective clinical trials, which combine age and biology to optimize the selection of patients who may not benefit from adjuvant radiation.

In the EBCTCG meta-analysis summarizing studies beginning before 2000, the rate of any first recurrence with BCS alone was 35% compared to 19.3% with BCS and RT, translating to a 10-year gain of 15.7% and a relative risk of 0.52 from RT [2]. In more modern series, the absolute local recurrence and the absolute benefit of whole breast RT are lower, at around 1.1–2% [9,12]. Therefore, improving the radiation delivery and toxicity has become increasingly important to maintain a favorable therapeutic ratio. 

Partial breast irradiation (PBI) involves focused radiotherapy delivery to the lumpectomy cavity rather than the whole breast. The rationale is that residual microscopic disease tends to lie within 2 cm of the initial tumor [13,14], and most local recurrences occur at or near the lumpectomy bed [15]. The UK IMPORT LOW study, which accrued patients between 2007 and 2010, evaluated outcomes after whole breast RT with 40 Gy in 15 fractions, reduced dose whole breast RT with 36 Gy in 15 fractions and 40 Gy in 15 fractions to the lumpectomy area, and partial breast RT with 40 Gy in 15 fractions [12]. The risk of local relapse at 6 years was 1.1%, 0.2%, and 0.5%, respectively. The toxicity of the two experimental arms with reduced dose and partial breast RT was lower, indicating that reducing RT fields does not compromise efficacy, nor does it increase side effects.

Accelerated RT involves higher doses delivered over a shorter interval of time. The rationale is that a shorter RT course with a higher dose per fraction can achieve the same therapeutic effect as a longer treatment course with a lower dose per fraction. A one-week schedule for whole breast or chest wall RT was evaluated in the UK FAST FORWARD study and found not to be inferior to standard fractionation without compromising normal tissue effects [16,17]. 

Accelerated partial breast irradiation (APBI) combines field reduction and acceleration of treatment time. APBI has been evaluated in phase III trials using percutaneous approaches, including multicatheter interstitial brachytherapy (MIB), balloon catheter intracavitary brachytherapy, electronic radiotherapy, and non-percutaneous approaches, including three-dimensional conformal external beam radiotherapy (3D-CRT), or intensity modulated radiation therapy (IMRT), proton beams or stereotactic PBI (Table 3). 

The first randomized controlled trial of APBI came from the Hungarian National Institute of Oncology for patients with T1, N0-1 mi, >40 years of age with negative surgical margins. Investigators reported 10-year IBTR rates of 5.9% and 5.1% in the APBI and WBI arms, respectively, using HDR MIB or an electron beam [18]. Subsequent clinical trials have demonstrated even lower IBTR rates of 2.5–4%, with mature follow-up at 8–10 years [19,20,21,22,23]. The Florence trial showed equivalent outcomes for WBI (50 Gy in 25 fractions with tumor bed boost) vs. APBI (30 Gy in five every-other-day fractions) using IMRT. There was a significantly lower rate of acute and late toxicity and improved cosmetic outcomes with APBI than with WBI at a median follow-up of 10 years [23]. The NSABP B-39/RTOG 0413 trial is the largest prospective study to date, with 4216 patients randomized to WBI (50 Gy in 25 fractions) vs. APBI via either multicatheter brachytherapy (34 Gy in 10 fractions BID), intracavity brachytherapy (MammoSite 34 Gy in 10 fractions BID), or 3D conformal radiation (3D-CRT) (38.5 Gy in 10 fractions BID). The 10-year cumulative incidence of IBTR was 4.6% in the APBI group versus 3.9% in the WBI group, hence not meeting the criteria for equivalence. However, the absolute difference in IBTR was <1%. Furthermore, the trial had broad eligibility criteria with heterogeneous APBI techniques. The study was not designed to test equivalence in patient subgroups or outcomes from different APBI techniques. Overall, MIB or single-entry brachytherapy or conformal EBRT appears to be most widely effective compared to intraoperative radiotherapy with regard to local control (Table 3).

Regarding toxicity and cosmesis, results have been mixed with a potential confounding effect from the radiation components of the trials. For one, the interfraction recovery kinetics impact tissue repair for late fibrosis is estimated to have a half-life of repair of 4.4 hours. The RAPID trial from Canada demonstrated non-inferiority using 3D-CRT (38.5 Gy in 10 fractions BID) with an interfraction interval of 6 hours [22]. This study found no difference in IBTR but an increase in moderate late toxicity and adverse cosmesis with APBI compared to hypofractionated WBI (42.5 Gy in 16 fractions). These findings suggest that perhaps 6 hours may have been insufficient for complete normal tissue repair. Furthermore, compared to the Florence trial in which 100% of patients were treated with IMRT, only 10% underwent IMRT in the RAPID trial, and the homogeneity and ipsilateral breast volume constraints were less strict.

A retrospective study of 345 patients treated at New York University (NYU) confirmed the low rates of toxicity and poor cosmesis with a five-fraction regimen [24]. In this study, 94% of patients were treated prone, with 32% treated every other day and 68% on consecutive days with 6 Gy × 5. The ipsilateral breast was constrained to V50% <60% and V100% <35%. 5-year IBTR rate was 1.8%. The rate of good or excellent physician- and patient-rated cosmesis (*n* = 199, median follow-up 2.8 years) was 92.5% and 89.4%, respectively. There were low rates of telangiectasia (4.5% grade 1 and 1.5% grade 2), fibrosis (17.6% grade 1 and 3.0% grade 2), and retraction/atrophy (24.1% grade 1, 2.5% grade 2, and 0.5% grade 3). Overall, a once-per-day EBRT-APBI regimen with a sufficient interval between fractions appears to have a significantly less adverse effect on cosmesis. A notable finding of the NYU retrospective review was that when comparing patients with IBTR to those without IBTR, a higher proportion of those who did not receive endocrine therapy experienced IBTR (67% vs 27%, *p* = 0.048). The NYU S14-0136 trial of prone APBI is a randomized controlled non-inferiority trial to compare radiation fibrosis with five (6 Gy × 5) versus three fractions (8 Gy × 3). The primary endpoint of the study is the rate of fibrosis at 2 years. The study closed accrual in June 2020, and we await the maturation of the data for the primary endpoint. 

The side effect profile of APBI over WBI in the modern era will likely continue to improve as we modify delivery schedules, volumes treated, and treatment techniques. The data indicate that APBI not only reduces the biological toxicity of radiotherapy but also reduces the financial toxicity, based on Medicare fee-for-service global reimbursement patterns. Future directions to continue improving the therapeutic ratio include increasing the use of conformal treatments with IMRT, the use of even smaller volumes with preoperative treatments, and additional research regarding dose-volume parameters that allow even safer delivery of treatment with regard to long-term toxicity. 

## 3. Emerging Data on Omission of Endocrine Therapy with Radiation Alone 

Adjuvant ET, which includes TAM, a selective estrogen receptor modulator, or AIs, is another variable in the multimodal treatment of early-stage HR+ breast cancer with the opportunity to explore de-escalation in select patients. Compliance with ET can be challenging, particularly in patients with comorbid conditions [25]. Rates of non-adherence in the literature are reported to be as high as 30–50% and were shown to be associated with increased mortality [26]. Non-adherence rates for TAM versus AIs are largely similar based on retrospective studies. There are a few studies that show slightly better adherence for AIs. However, a meta-analysis reported a 5-year discontinuation rate for TAM to be 47.2% and AI to be 31.0% [27], noting that non-adherence rates are not insignificant with AI. 

All the radiation omission trials required ET. In the CALGB 9343 and PRIME II trials, the omission of radiotherapy discontinuation of ET rates were not reported, clouding our interpretation of results, and limiting the generalizability of the study findings. In a meeting for a multi-hospital retrospective review of 115 patients, the criteria for the LUMINA trial from the US, the ET non-adherence rate, defined as discontinuation before 5 years due to reasons unrelated to disease recurrence or death, was compared between women treated with RT versus RT omission (PD7-04, San Antonio Breast Cancer Symposium 2021). ET non-adherence was found to be 32% in the RT omission group versus 17% in the RT group. The rate of locoregional control was reduced to 83% from 100% in the RT omission groups among ET adherent versus non-adherent patients (*p* = 0.18). Overall, while local control with ET alone is excellent for patients who are adherent, the same may not be true for non-adherent patients. It is plausible that patients who opt for RT omission may also be more likely to have poor ET compliance for other medical or psychosocial reasons and personal preferences in their healthcare choices. 

With APBI and the UK FAST FORWARD accelerated whole breast regimen, the question remains: does shortening of RT treatment duration and field alter the balance between the optimal adjuvant strategies for early-stage breast cancer patients? Is APBI superior in terms of health-related quality of life compared to ET alone? A SEER Medicare study of 13,321 women aged 66–85, diagnosed with stage I ER+ breast cancer from 2006–2012, with continuous enrollment in Medicare Parts A, B, and D with claims through 2014 patients with lumpectomy and more than 2 years of follow-up were analyzed [28]. There were four groups according to treatment: ET + RT (43.5%), ET alone (6.6%), RT alone (41.3%), and neither ET nor RT (8.6%). At a median follow-up time of 43 months, it was found that lower comorbidity, higher T stage, and younger age were associated with a higher likelihood of undergoing ET+RT than other strategies. There was an increased risk of a second breast cancer event with no treatment or ET alone but not with RT alone, suggesting that RT alone may, in fact, be a reasonable strategy for de-escalation. The EUROPA trial (NCT04134598), which began accruing patients in early 2021, is currently investigating the omission of ET for women ≥70 years with early-stage breast cancer. The primary outcome measures are patient-reported health-related quality of life and time to IBTR. Assuming an equal rate of disease control, unnecessary long-term toxicity of ET may be avoided with this approach. 

Employing gene signatures based on multigene profiling assays are potential tools to select patients for whom omission of ET may be the best strategy. The 70-gene MammaPrint gene expression assay (Agendia), which has an NCCN Category 1 rating in breast cancer for pN0 and 1–3 positive nodes, can identify a subgroup of patients (MammaPrint Index (MPI) > +0.355) as ultralow risk who have an excellent 20-year survival risk without any systemic therapy. The Stockholm tamoxifen (STO-3) study was a randomized clinical trial of TAM versus no therapy from 1976 until 1990 in postmenopausal women with node-negative, ER-positive breast cancer and tumors <3 cm who had received either adjuvant TAM for two years or no systemic therapy and were randomized to receive three additional years of TAM or no further treatment [29]. Sixty-five percent of patients in the study received TAM for only two years. In a secondary analysis of FFPE tissue blocks, 15% of patients were categorized as ultralow risk using MammaPrint. The 20-year DFS in these patients was 97% in the TAM group and 94% in the no TAM group, suggesting that the ultralow-risk threshold could identify patients whose long-term risk of dying from breast cancer is exceedingly low, despite no or limited ET [27]. 

In a subset analysis of the prospective MINDACT trial [30], 1000 patients were identified as an ultralow risk by MammaPrint (15% of the total population) [31]. Among the ultralow-risk patients, 67% were over the age of 50, 81% had tumors <2 cm, 80% were lymph node-negative, 99% were ER-positive, and 96% had tumors that were grade 1 or 2. Eighty-three percent of patients received systemic therapy (69% with ET and 14% with ET + chemotherapy), and 16% of these patients received no adjuvant systemic therapy. Breast cancer-specific survival and distant metastasis-free survival (DMFS) for this group at 8 years was 99.6% (95% CI 99.1–100) and 97% (95% CI 95.8–98.1), respectively. Among the patients who received no adjuvant systemic therapy, the DMFS at 8 years was 97.8% (95% CI 95.3–100). 

The Breast Cancer Index (BCI) is a gene expression-based biomarker developed using a cohort of ER+ lymph node-negative patients from the randomized prospective Stockholm trial using two biomarkers, the HOXB13:IL17BR ratio (H/I) and the molecular grade index (MGI) [32]. Investigators reported that a high H/I ratio could predict the TAM benefit, whereas H/I-low patients did not benefit in patients in a multi-institutional cohort. Decreasing the duration of ET is another de-escalation strategy under investigation. 

APBI alone and ET alone are potentially reasonable monotherapy strategies for patients with very low-risk breast cancer. In the Florence APBI trial of five fractions, it is notable that 35.8% of patients in the APBI arm and 28.8% in the WBI arm did not receive any adjuvant ET. Cost-effective analyses have revealed that both APBI alone and ET alone are appropriate options for patients older than 70 years of age with small cost differences [33]. While APBI is largely comparable in cost to 5 years of AI alone, when AI compliance is lower than 26% at 5 years, APBI becomes more cost-effective than AI alone. 

## 4. Conclusions

In HR+ stage I breast cancer, older age combined with tumor subtype identifies patients at lower risk of local recurrence in whom the absolute benefit of both adjuvant radiotherapy and endocrine therapy remains small. Randomized trial evidence exists to support the omission of radiation based on age and tumor subtype, all of which required endocrine therapy. We are awaiting trials combining age and tumor biology, including utilizing multigene assays, to identify patients for whom omission is reasonable. De-escalation of radiotherapy in the form of reduced treatment fields and accelerated treatment time maintains a favorable therapeutic ratio, even with a decrease in the absolute benefit of radiation on local control in the modern era. There is emerging data on the omission of endocrine therapy when radiation is given in a low-risk patient population, and we await prospective trials to identify appropriate patients for endocrine therapy omission or de-escalation. Much progress has been made in reducing the overall treatment burden for early-stage breast cancer patients while maintaining excellent outcomes, and we look forward to continuing to refine the optimal treatment strategy for these patients.

## Figures and Tables

**Table 1:** Clinical trials on the omission of radiation therapy in early-stage breast cancer.

Author/Study	Dates of Accrual	Final Date of Publication	Patients	Randomization	Radiotherapy	Age	Tumor Subtype	Tumor Size	IBTR	Notable Results
Fisher et al. (NSABP B21)	1989–1998	2002	1009 patients’ status post lumpectomy	TAM vs. XRT vs. XRT + TAM	50 Gy in 2 Gy fractions, optional boost (median dose 10 Gy)	20% <50 years old; 16% >70 years old	56.7% ER+	All patients ≤1 cm (27.7% ≤ 5 mm)	At 8 years, TAM: 16.5%; XRT: 9.3%; XRT + TAM: 2.8%	Significant reduction in contralateral breast cancer with tamoxifen; OS in 3 groups were equivalent at 93–94%
Fyles et al.	1992–2000	2004	769 women with pT1-2N0 breast cancer status post-lumpectomy	TAM vs. XRT + TAM	40 Gy in 2.5 Gy fractions, 12.5 Gy boost in 2.5 Gy fractions, 16 treatments (3–4 weeks)	All patients ≥50 years old; 42% ≥70 years old	80.8% HR+	34.7% ≤ 10 mm	At 8 years, TAM: 17.6% vs. TAM + XRT: 3.5%	Planned subgroup analysis of T1, HR+ (*n* = 611) with 8-year IBTR of 15.2% vs. 3.6%. Unplanned subgroup analysis of women >60, tumors <1 cm (*n* = 193), HR+ with 8 year IBTR 3.6% vs. 0%
Hughes et al. (CALGB 9343)	1994–1999	2013	636 women with T1N0M0 breast cancer status post lumpectomy	TAM vs. XRT + TAM	45 Gy in 1.8 Gy fractions, electron boost of 14 Gy in 2 Gy fractions, 32 treatments (6.5 weeks)	All patients ≥70; 55% ≥75	99% ER+	98% ≤ 20 mm	At 10 years, 98% in XRT + TAM vs. 90% in TAM arm were free from LRR	LRR benefit did not translate into an advantage in OS, DFS, or breast preservation
Kunkler et al. (PRIME II)	2003–2009	2015	1326 women with node-negative breast cancer status post lumpectomy	XRT vs. no XRT	40–50 Gy in 2.66–2.00 Gy per fraction, allowance of 10–15 Gy electron boost or iridium implant, 15–25 treatments	All patients ≥65	All patients ER+, PR+, or both.	Tumors up to 3 cm (39.4% ≤10 mm)	At 5 years, XRT: 1.3% vs. no XRT: 4.1%; at 10 years, XRT: 0.9% vs no XRT: 9.8%	There were no differences in regional recurrence, contralateral breast cancer, or distant metastases. Most patients dying of causes unrelated to breast cancer.

Tam = Tamoxifen; XRT = Radiation therapy; ER+ = Estrogen receptor positive; OS = Overall survival; HR+ = Hormone receptor-positive; IBTR = Ipsilateral breast tumor recurrence; LRR = Locoregional recurrence.

**Table 2:** Ongoing clinical trials for the omission of radiation therapy incorporating multigene assays.

Trial	Clinicaltrials.gov Identifier	Study Start Date	Estimated Study Completion Date	Study Design	Age	Molecular Subtype	Grade	Biological Selection	Target Accrual	Recruitment Status	Preliminary Results
LUMINA	NCT01791829	July 2013	December 2024	Phase II, single-arm observational	>50 years	ER and PR+ in ≥10%; HER2- on IHC (0 or 1+) or FISH	Grade 1 or 2	Luminal A subtype by IHC	500	Active, not recruiting	ASCO 2022: at 5 years, 10 local recurrences (2.3%) and 8 contralateral breast cancer events (1.9%); OS 97.2%, DFS 89.9%, RFS 97.3%
IDEA	NCT02400190	March 2015	March 2026	Phase II, single-arm observational	50–69 years	ER+, PR+, Her2- using the current College of American Pathologists guidelines	-	Oncotype DX RS ≤18	250	Active, not recruiting	-
PRECISION	NCT0265375	May 2016	December 2025	Phase II, single-arm observational	50–75 years	ER+ (≥ 10%) or PR+, HER2-	Grade 1 or 2	Prosigna PAM-50, ROR ≤40	671 screened, 382 study population	Active, not recruiting	-
EXPERT	NCT02889874	August 2017	December 2023	Phase III Randomized trial RT vs observation	≥50 years	ER and PR+ in ≥10%; HER2- on IHC (0 or 1+) or FISH	Grade 1 or 2	Prosigna PAM-50, ROR ≤60	1167	Active, recruiting	-
DEBRA	NCT04852887	June 2021	July 2024	Phase III Randomized trial RT vs observation	50–<70 years	ER≥1%, PR+, Her2- using ASCO/CAP Guideline Recommendations	-	Oncotype DX RS ≤18	1670	Active, recruiting	-

ER+ = Estrogen receptor positive; PR+ = Progesterone receptor positive; HER2- = Human epidermal growth factor receptor 2 negative; IHC = Immunohistochemistry; OS = Overall survival; DFS = disease-free survival; RFS = Recurrence-free survival; RS = Recurrence score; PAM-50 = Prediction analysis of microarray 50; ROR = Risk of recurrence.3. Radiation Acceleration and De-Escalation

**Table 3:** Clinical trials of partial breast irradiation.

Author/Study	Country	Dates of Accrual	Final Date of Publication	Patients	PBI	WBI	Boost in WBI Arm	Follow up	IBTR	Toxicity and Cosmesis
Polgar et al.	Hungary	2006–2019	2013	258 patients with pT1 (≤ 2 cm), pN0–1 mi, negative margins, age >40	36.4 Gy/7 fx (HDR) or 50 Gy/25 fx (electrons)	50 Gy/ 25 fx	16 Gy electrons (0.8%)	10.2 years	APBI: 5.9% vs. WBI: 5.1%	APBI had higher excellent-good cosmetic score (81% vs. 63%)
Rodriguez et al.	Barcelona	N/A	2013	102 patients with pT1–2 (≤3 cm), pN0, margins ≥ 2 mm, age ≥ 60	37.5 Gy/10 fx BID (3D-CRT)	48 Gy/24 fx	10 Gy (66.0%)	5 years	APBI: 0% vs. WBI: 0%	No difference in late skin toxicity or cosmesis
Strnad et al. (GEC-ESTRO)	Austria, Czech Republic, Germany, Hungary, Poland, Spain, and Switzerland	2004–2009	2016	1184 patients with pT1–2 (<3 cm), pN0–1 mi, margins ≥ 2 mm, age ≥ 40	32 Gy/8 fx or 30.2 Gy/7 fx (HDR) or 50 Gy (PDR)	50 Gy/25 fx	10 Gy electrons (100.0%)	6.6 years	APBI: 1.4% vs. WBI: 0.92% (p=0.42)	Significantly lower grade 2+ late skin effects with APBI
Coles et al. (IMPORT LOW)	United Kingdom	2007–2016	2017	2018 patients with pT1-2 (<3 cm), N0–1, margins ≥ 2 mm, age ≥ 50	40 Gy/15 fx	40 Gy/15 fx vs 36 Gy + 40 Gy boost	Simultaneous integrated boost in 36 Gy+40 Gy arm	6 years	PBI: 0.5% vs. WBI: 1.1% vs. reduced dose WBI + boost: 0.2%	Reduced toxicity in both experimental arms
Vicini et al. (NSABP B-39)	USA, Canada, Ireland, and Israel	2005–2018	2019	4216 patients with pT1–2 (<3 cm), pN0–1 (1–3), negative margins, age ≥ 18	38.5 Gy/10 fx BID (3D-CRT) or 34 Gy/10 fx (HDR)	50 Gy/ 25 fx WBI	10–16 Gy (80%)	10.2 years	APBI: 4.6% vs. WBI: 3.9% (HR did not meet criteria for equivalence)	In non-chemotherapy treated patients, APBI had slightly poorer cosmesis at 3 years
Whelan et al. (RAPID)	Canada	2006–2918	2019	2135 patients with pT1–2 (≤ 2 cm), pN0–1 mic, negative margins, age ≥ 40	38.5 Gy/10 fx BID (3D-CRT)	42.5 Gy/ 16 fx (82%), 50 Gy/25 fx (18%)	10 Gy (21%)	8.6 years	APBI: 3% vs. WBI: 2.8% (HR met criteria for equivalence)	APBI had less acute and more late toxicity (grade 2+), similar patient-rated cosmetic outcome
Livi et al. (Florence)	Italy	2005–2013	2020	520 patients with pT1–2 (<2.5 cm), negative margins, age > 40	30 Gy/5 fx once-daily, non-consecutive days	50 Gy / 25 fx	10 Gy electrons (100%)	10.7 years	APBI: 3.7% vs WBI: 2.5% (*p* = 0.40)	APBI had less acute and late toxicity and improved patient and physician rated cosmetic outcome
Vaidya et al. (TARGIT)	United Kingdom, Europe, Australia, the United States, and Canada	2000–2012	2020	2298 patients, <3.5 cm, cN0-N1, age >45	20 Gy IORT	3–6 weeks EBRT	optional boost	5 years	IORT: 2.11% vs WBI: 0.95%	Grade 3 or 4 radiotherapy toxicity was significantly reduced with TARGIT
Orrechia et al. (ELIOT)	Italy	2000–2007	2021	1305 patients, <25 mm, cN0, age 48–75 years	21 Gy IORT	50 Gy/ 25 fx	10 Gy (100%)	12.4 years	IORT: 11% vs WBI: 2%	-
ASTRO APBI Guidelines				pT1 (≤ 2 cm), pN0–1 mi, margins ≥ 2 mm, age ≥ 50; DCIS: screen-detected, 1–2 nuclear grade, ≤2.5 cm size, margins ≥ 3 mm						

PBI = Partial breast irradiation; WBI = Whole breast irradiation; IBTR = Ipsilateral breast tumor recurrence; fx = fractions; APBI = Accelerated partial breast irradiation; 3D-CRT = Three-dimensional conformal radiotherapy; HDR= high dose rate; PDR = Pulsed dose rate; IORT = Intraoperative radiation therapy; DCIS = Ductal carcinoma in situ.

## Data Availability

Not applicable.
